# The Transition From Stochastic to Deterministic Bacterial Community Assembly During Permafrost Thaw Succession

**DOI:** 10.3389/fmicb.2020.596589

**Published:** 2020-11-13

**Authors:** Stacey Jarvis Doherty, Robyn A. Barbato, A. Stuart Grandy, W. Kelley Thomas, Sylvain Monteux, Ellen Dorrepaal, Margareta Johansson, Jessica G. Ernakovich

**Affiliations:** ^1^Department of Molecular, Cellular, and Biomedical Sciences, University of New Hampshire, Durham, NH, United States; ^2^Cold Regions Research and Engineering Laboratory, Engineer Research Development Center, United States Army Corps of Engineers, Hanover, NH, United States; ^3^Department of Natural Resources and the Environment, University of New Hampshire, Durham, NH, United States; ^4^Department of Soil and Environment, Swedish University of Agricultural Sciences, Uppsala, Sweden; ^5^Climate Impacts Research Centre, Department of Ecology and Environmental Sciences, Umeå University, Abisko, Sweden; ^6^Department of Physical Geography and Ecosystem Science, Lund University, Lund, Sweden

**Keywords:** permafrost thaw, microbial community, community assembly, phylogenetic null modeling, ecological processes

## Abstract

The Northern high latitudes are warming twice as fast as the global average, and permafrost has become vulnerable to thaw. Changes to the environment during thaw leads to shifts in microbial communities and their associated functions, such as greenhouse gas emissions. Understanding the ecological processes that structure the identity and abundance (i.e., assembly) of pre- and post-thaw communities may improve predictions of the functional outcomes of permafrost thaw. We characterized microbial community assembly during permafrost thaw using *in situ* observations and a laboratory incubation of soils from the Storflaket Mire in Abisko, Sweden, where permafrost thaw has occurred over the past decade. *In situ* observations indicated that bacterial community assembly was driven by randomness (i.e., stochastic processes) immediately after thaw with drift and dispersal limitation being the dominant processes. As post-thaw succession progressed, environmentally driven (i.e., deterministic) processes became increasingly important in structuring microbial communities where homogenizing selection was the only process structuring upper active layer soils. Furthermore, laboratory-induced thaw reflected assembly dynamics immediately after thaw indicated by an increase in drift, but did not capture the long-term effects of permafrost thaw on microbial community dynamics. Our results did not reflect a link between assembly dynamics and carbon emissions, likely because respiration is the product of many processes in microbial communities. Identification of dominant microbial community assembly processes has the potential to improve our understanding of the ecological impact of permafrost thaw and the permafrost–climate feedback.

## Introduction

Permafrost, soil that has been frozen for two or more consecutive years, underlies approximately one fourth of the northern hemisphere (Zhang et al., 1999) and is undergoing thaw with increasing global temperature (Romanovsky et al., 2017). The Northern high latitudes are experiencing warming twice as fast as the global average (Overland et al., 2019) with an expected increase of 5°C to 6°C in the surface air temperature by the end of this century (Stocker et al., 2014). It is estimated that permafrost stores approximately 1300 Pg of carbon which is equal to that found in Earth’s atmosphere and above and belowground vegetation combined (Hugelius et al., 2014). Following thaw, soil microorganisms decompose this carbon resulting in the release of greenhouse gases such as carbon dioxide (CO_2_), methane (CH_4_), and nitrous oxide (N_2_O) to the atmosphere (Schädel et al., 2014; Treat et al., 2016; Voigt et al., 2017). As a result, these gases create a positive feedback to global warming, further threatening permafrost degradation.

Permafrost thaw induces changes to microbial community composition and functional potential (Mackelprang et al., 2011; Coolen and Orsi, 2015; Hultman et al., 2015). In microbial systems, dramatic disturbances of the local environment can lead to mass extinction and essentially “reset” a community’s trajectory (Ferrenberg et al., 2013). Simulated permafrost thaw experiments conducted in a controlled laboratory environment have shown that thawing over relatively short time scales (e.g., days to months) results in different microbial community structure than before thaw (Mackelprang et al., 2011; Ernakovich et al., 2017). Field thaw experiments indicate that permafrost community compositions shift over longer time scales to resemble active layer communities along depth profiles (Deng et al., 2015; Mondav et al., 2017; Monteux et al., 2018). Functional shifts have also been observed during permafrost thaw. Frozen conditions promote genes involved in stress responses and survival strategies, and thaw results in increases in genes involved in decomposition of soil organic matter and transport of soil nutrients (Mackelprang et al., 2011, 2017; Coolen and Orsi, 2015; Hultman et al., 2015).

Since soil microorganisms regulate many important biogeochemical processes, such as carbon and nitrogen cycling, it is critical to understand how microbial communities are shaped during permafrost thaw and to what degree this will affect ecosystem level processes. Microbial community structure is important for ecosystem processes (Graham et al., 2016), but to what degree it matters depends on the physical and phylogenetic scale in question (Schimel, 1995). The structure of microbial communities is influenced by both deterministic and stochastic ecological assembly processes. Deterministic processes are driven by abiotic and biotic selection pressures that influence the fitness of a population in a given environment (Vellend, 2010; Nemergut et al., 2013). Stochastic processes, which include inherent randomness, are less predictable and include diversification (genetic variation), drift (random changes in species abundances), and dispersal (movement of species across space) (Vellend, 2010; Nemergut et al., 2013). Many microbiome studies attribute patterns of community structure only to deterministic processes (reviewed in Zhou and Ning, 2017). However, stochastic processes play an important role in structuring communities that is underappreciated in microbial ecology due to difficulty in defining stochastic processes and the variety of approaches used to assess stochasticity (Nemergut et al., 2013; Zhou and Ning, 2017). When communities are shaped by deterministic processes, variations in the local environment may directly influence functional outcomes since microbial traits are selected for by environmental conditions. Alternatively, when communities are structured by stochastic processes, function may be dependent on random shifts in trait abundances within the community, rather than a direct relationship with the environment (Knelman and Nemergut, 2014).

Assembly processes in permafrost systems provide important insights into drivers of microbial community structure in intact and thawed conditions. Bottos et al. (2018) found that bacterial communities in intact permafrost are structured by dispersal limitation. The permafrost environment is also thought to be selective for organisms that can survive subzero temperatures for extended periods of time (reviewed in Jansson and Taş, 2014), suggesting deterministic processes may also play a large role. Increases in soil temperature due to either experimental warming or long-term permafrost thaw have resulted in an increase of deterministic processes structuring active layer microbial communities (Mondav et al., 2017; Feng et al., 2020). Furthermore, Tripathi et al. (2018, 2019) found stochastic processes dominate community assembly in deeper soils compared to surface soils in permafrost systems. However, these studies lack a direct comparison of assembly processes in permafrost soils pre- and post-thaw. Upon thaw, the local environment changes dramatically and may present a physiological challenge for these microbes that have become accustomed to living in permafrost conditions. Immediately after a disturbance, assembly processes are more stochastic likely due to mass extinction events leading to ecological equivalence of individuals and immigration with little competition (Ferrenberg et al., 2013; Dini-Andreote et al., 2015). As succession progresses, selection begins to play a large role in structuring communities (Ferrenberg et al., 2013; Dini-Andreote et al., 2015). Therefore, we speculate that microbial communities in newly thawed permafrost are likely structured by stochastic processes, but as time since thaw progresses there is a shift toward deterministic assembly. In order to develop a robust framework to predict permafrost community dynamics following thaw, laboratory and field studies are needed to characterize shifts in microbial community assembly during thaw in both active layer and permafrost soils. Since outcomes of stochastic assembly may be more difficult to predict due to inherit randomness, the immediate implications of permafrost thaw may be difficult to understand if stochastic assembly plays a large role in structuring post-thaw communities.

The overall objective of this study was to determine the relative contribution of stochastic and deterministic assembly processes in active layer and permafrost soils pre- and post-thaw. Specific objectives were to characterize the effect of time since thaw on assembly processes and evaluate the effect of increased temperature on assembly dynamics and microbial functions. We used the soil depth profile as a proxy of “time since thaw” by incorporating changes in active layer thickness over a fourteen-year period at the Storflaket Mire in Abisko, Sweden. We hypothesized that the uppermost active layer and permafrost communities would be dominated by environmental selection and transition zone communities would be dominated by stochastic assembly due to recent shifts in the abiotic environment. To test the direct effect of increased temperature on assembly dynamics we subjected soil samples from four depths along the depth profile to incubation at 4°C and 15°C. We hypothesized community assembly would be more stochastic at all depths after lab-induced thaw compared to *in situ* assembly. Specifically, the warmer incubation temperature leading to the most stochastic assembly due to the higher temperature representing a greater disturbance to the community. Comparing assembly patterns between field and lab thaw scenarios will elucidate the potential differences in community assembly at long versus short timescales after thaw.

## Materials and Methods

### Site Description, Sample Collection, and Processing

**FIGURE 1:**
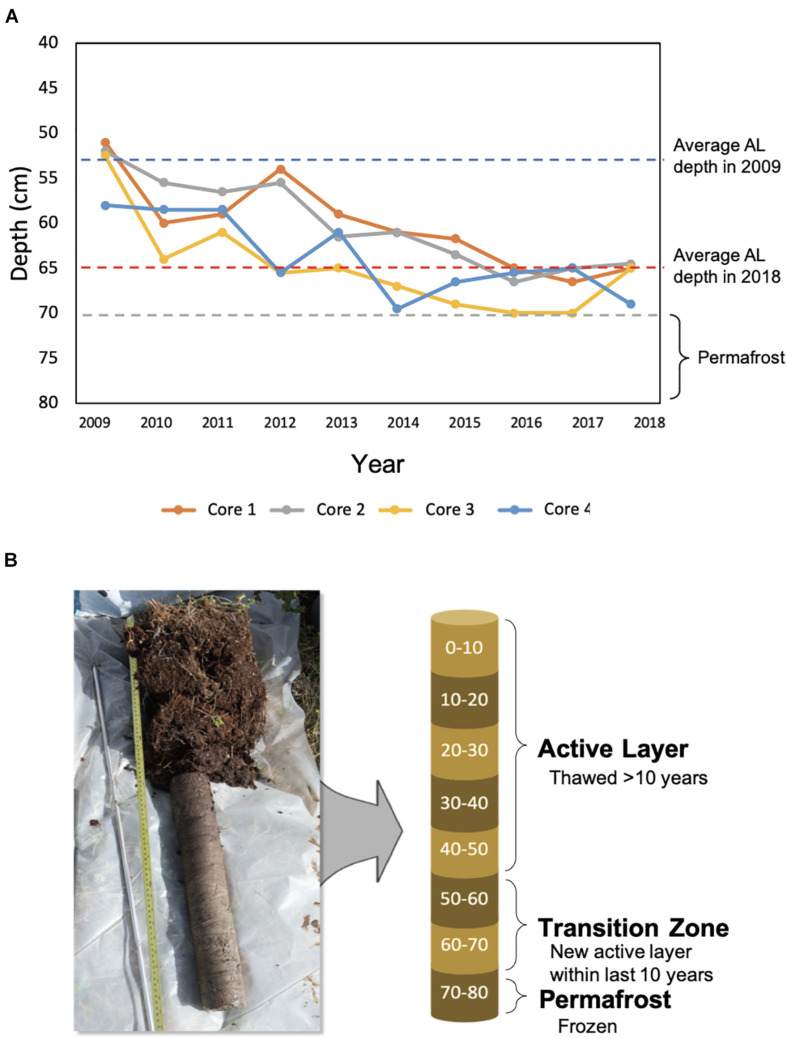
Changes in active layer thickness and core subsampling. **(A)** The active layer thickness at sampling locations from 2009 to 2018. In 2009, the average active layer thickness across locations was 53 cm, and in 2018, it was 65 cm. Active layer thickness did not exceed 70 cm during this time period. **(B)** Each core was subsampled every 10 cm. Approximate time since thaw was used to differentiate active layer, transition zone, and permafrost soils.

Permafrost and active layer soil samples were collected from control plots used in a snow manipulation experiment (Johansson et al., 2013) at the Storflaket Mire in Abisko, Sweden (68°20′48″N, 18°58′16′E). Active layer thickness measurements were recorded across experimental plots during peak thaw in September each year resulting in a detailed record of active layer thickness over a fourteen-year period. A total of four replicate cores were collected from control plots at the site. Sampling locations were chosen to ensure similar active layer thickness and thaw histories were captured in the replicate cores (Figure 1A). At the time of sampling in June 2019, active layer soils had only thawed to approximately 34 cm. The permafrost began at 65 cm according to the 2018 active layer thickness data that was used to estimate the permafrost depth.

The thawed active layer was collected using a sterile serrated knife cleaned with 70% ethanol, DNA Away, and RNAse Away solutions (Thermo Fisher Scientific, Waltham, MA, United States) and set aside on a clean plastic tarp. The frozen active layer and permafrost were collected using a SIPRE corer (Jon’s Machine Shop, Fairbanks, AK, United States) fitted with a gas-powered motor. The soil core was reconstructed by laying each piece on a cleaned plastic tarp. The thawed active layer was sub-sectioned into 10 cm sections up to 30 cm (0–10 cm, 10–20 cm, 20–30 cm) and placed in sterile Whirlpak bags. Frozen active layer and permafrost samples were bagged intact and sub sectioned back in the lab for the remaining depths: 30–40 cm, 40–50 cm, 50–60 cm, 60–70 cm, and 70–80 cm. All samples were stored on ice during transit.

All soil samples were subsampled for DNA analysis and soil incubation in a 2°C cold room at the Abisko Scientific Research Station. Tyvek suits and face masks were worn to reduce potential contamination of samples. All tools were sterilized with 70% ethanol, DNA Away, and RNAse Away solutions (Thermo Fisher Scientific, Waltham, MA, United States). Frozen cores were subsampled every 10 cm by cutting through the core with a sterile wire hand saw and broken apart with a mallet and sterile chisel. From each 10 cm segment, three smaller pieces were taken for DNA analysis of the *in situ* (“initial”) community using a sterile hole saw bit and electric drill. All samples were stored at −20°C until shipment. Samples used to assess initial communities were hand carried on dry ice during transit and stored at −80°C upon arrival. Remaining samples to be used in the soil incubation thaw experiment were shipped frozen to the University of New Hampshire (UNH) and thawed to room temperature during the 12 days in transit. Upon arrival at UNH, these samples were stored at 4°C for 3 days prior to incubation set up.

### Abiotic Analyses

Physical and chemical properties were characterized for “initial” samples using the thawed soils. Gravimetric water content (GWC), pH, total combustible carbon and nitrogen were measured. Soil GWC was determined by weighing approximately 10–20 g of soil from each biological replicate and drying at 105°C to a constant mass for 24 h. GWC was calculated on a per dry mass basis. Soil pH was determined by shaking a slurry of fresh soil and water (1:10) for 1 h and measured using an Accumet basic AB 15 pH meter (Thermo Fisher Scientific, Waltham, MA, United States). Soil was air dried and ground to a fine powder using a ball mill grinder fitted with plastic inserts. Ground soils were analyzed for total combustible carbon and nitrogen via thermal oxidation with gas chromatographic separation followed by thermal conductivity detection on a Costech C/H/N/S Elemental Analyzer (Costech Analytical Technologies, Inc., Valencia, CA, United States).

### Soil Incubation

A soil incubation experiment was conducted to assess microbial community assembly after lab-induced thaw. The soil incubation was conducted at two different thaw temperatures. The 4°C temperature was chosen to reflect near-term thaw conditions and the 15°C temperature emulated a more drastic thaw state representative of the predicted temperature increase for the end of this century at northern high latitudes (Stocker et al., 2014). Four depths from each core were chosen to represent upper active layer (10–20 cm), lower active layer (30–40 cm), transition zone (50–60 cm), and intact permafrost (70–80 cm). For each depth, two subsamples of approximately 40 g of fresh soil were weighed into ethanol-cleaned specimen cups and placed inside a liter sized glass jar with deionized water at the bottom to maintain moisture content (Supplementary Figure S1). Lids were fitted with valves to allow for headspace gas analysis to monitor heterotrophic respiration throughout the incubation. Subsamples were preincubated in a controlled incubator in the dark at either 4°C or 15°C for 5 days. After the preincubation, the jars were flushed with CO_2_-free air (Airgas, Dover, NH, United States) for 10 min. Jars were then sealed and incubated at either 4°C or 15°C in the dark. Individual cores served as replicates for this experiment (4 depths × 2 temperatures × 4 replicates (cores) = 32 samples).

Headspace gas measurements were analyzed every 2–7 days to ensure CO_2_ concentrations remained below 2% and oxic. Jars were removed individually from the incubator and attached to a Picarro G2201-i cavity ring down spectrometer (Picarro, Santa Clara, CA, United States). Once readings stabilized, the CO_2_ concentration was recorded, and the jar was flushed with CO_2_-free air (Airgas, Dover, NH, United States) for 10 min. The spectrometer was calibrated using gas standards prepared by mixing a known volume of CO_2_ calibration gas and CO_2_-free air. Standards were generated to span the operational range of the instrument 100–4,000 ppm CO_2_. Methane concentrations did not exceed the lower limit of the operational range of the analyzer (1.8 ppm CH_4_) at any point during the incubation. Respiration rate was calculated to μg C-CO_2_ g^–1^ dry soil h^–1^ (Equation 1). Dry weight was calculated using the gravimetric water content of the samples.

$$\mu g{\text{C-CO}}_{2\,}g^{-1}\mathrm{dry}\mathrm{soil}h^{-1}=\frac{{\text{CO}}_{2\,}\,\mu \,m\,o\,l}{\mathrm{mol}\,\mathrm{air}}\times$$

$$P\times V\times \frac{1}{R}\times \frac{1}{T}\times \frac{1}{g\,\mathrm{dry}\,\mathrm{soil}}\times \frac{12\,\mu \,g\,C}{1\,\mu \,\mathrm{mol}\,C}\times \frac{1}{t}$$

*Equation 1*. Respiration rate calculation where *P* is atmospheric pressure in atm, *V* is headspace volume in L, *R* is ideal gas constant in L atm K^–1^ mol^–1^, *T* is incubation temperature in K, and *t* is incubation length in hours.

Temperature sensitivity (*Q*_10_) was also calculated to investigate the effect of warming on carbon processing rates, as measured by CO_2_ production (Equation 2). *Q*_10_ values indicate the factor by which respiration increases when temperature is increased by 10°C.

$$Q_{10}={\left(\frac{k_{15}}{k_{4}}\right)}^{\left(\frac{10}{15-4}\right)}$$

*Equation 2*. Temperature sensitivity (*Q*_10_) calculation where *k*_15_ and *k*_4_ are respiration rates at 15°C and 4°C, respectively.

Samples were harvested after 193 days of incubation (“post-thaw”) to determine microbial community composition and assembly processes at the two incubation temperatures.

### Microbial Community Analysis

Genomic DNA was extracted from both “initial” and “post-thaw” incubation experiment samples and sequenced to assess the microbial community composition and dominant assembly mechanisms. Frozen samples were homogenized prior to analysis using a mallet to crush large chunks. DNA was extracted using the Qiagen DNeasy PowerSoil kit (Qiagen, Hilden, Germany) with minor changes to manufacture’s protocol (Supplementary Material). Each sample was extracted in triplicate and loaded onto the same spin filter to concentrate the DNA to increase yield in the permafrost samples which were expected to have low biomass. A MoBio PowerClean kit (MoBio, Carlsbad, CA, United States) was used to remove PCR inhibitors from the extracted DNA. DNA was then quantified using the Quant-iT dsDNA High Sensitivity Assay Kit and Qubit 3.0 fluorometer (Invitrogen, Carlsbad, CA, United States).

DNA was amplified through polymerase chain reaction (PCR) using the primers 515f-806r of the V4 region of the 16S rRNA gene to profile the bacterial and archaeal communities (Apprill et al., 2015; Parada et al., 2016) and the primers ITS1f-ITS2 of the internal transcribed spacer to profile the fungal community (White et al., 1990) (Supplementary Table S1). The reactions were performed separately for the two primer sets as follows. Each 16S rRNA reaction contained 6 μL DreamTaq Hot Start Green (Thermo Fisher Scientific, Waltham, MA, United States), 2.6 μL sterile water, 0.7 μL forward primer (5 μM), 0.7 μL reverse primer (5 μM), and 2 μL template DNA (10× diluted). Each ITS reaction contained 6 μL DreamTaq Hot Start Green, 3 μL sterile water, 0.5 μL forward primer (5 μM), 0.5 μL reverse primer (5 μM), and 2 μL template DNA (10× diluted). Amplifications were performed using a T100 Thermal Cycler (Bio-Rad, Hercules, CA, United States). The 16S rRNA conditions were: enzyme activation at 94°C for 3 min, followed by 35 cycles of denaturation at 94°C for 45 s, annealing at 50°C for 60 s, and extension at 72°C for 90 s, followed by final extension at 72°C for 10 min. The ITS conditions were: enzyme activation at 95°C for 3 min, followed by 35 cycles of denaturation at 95°C for 30 s, annealing at 52°C for 30 s, and extension at 72°C for 60 s, followed by final extension at 72°C for 12 min. No template controls were included to verify there was no contamination during the PCR. The presence of PCR product was confirmed through gel electrophoresis and quantified using the Qubit 3.0 fluorometer. PCR product concentration ranged from 2–27 ng/μL with deeper soil samples having lower concentrations than the near surface soil samples. PCR products were sent to the Hubbard Center for Genomic Studies (University of New Hampshire, NH, United States) for sequencing by Illumina HiSeq2500 with Rapid Run© SBS V2 chemistries (Illumina, San Diego, CA, United States) and 2 × 250 bp paired-end reads. Reads were demultiplexed using CASAVA (version 1.8; Illumina, San Diego, CA, United States).

Sequences were analyzed using QIIME 2 (version 2019.4) (Bolyen et al., 2019) on the Premise high performance computing cluster (University of New Hampshire, NH, United States). Primers were removed using Cutadapt (Martin, 2011) and then quality filtered with DADA2 (Callahan et al., 2016). For ITS analysis, ITSxpress (Rivers et al., 2018) was used to remove conserved regions to improve taxonomic classification (Nilsson et al., 2009). Taxonomy was assigned to amplicon sequence variants (ASVs) using scikit-learn naïve Bayes taxonomy classifier (Pedregosa et al., 2011) against the SILVA 99% database (Quast et al., 2012) for bacteria and UNITE database (Nilsson et al., 2019) for fungi. ASVs were filtered to remove chloroplast, mitochondria, and ASVs without phylum level classification. Bacteria and archaea were split into separate ASV tables. Due to low sequencing depth of archaea, it was not analyzed further in this study. ASVs were aligned with MAFFT (Katoh and Standley, 2013) and used to construct a phylogeny with FastTree2 (Price et al., 2010). To assess community composition along the depth profile, samples were rarefied to 2500 sequences per sample for bacteria and 950 for fungi. For the incubation study, samples were rarefied to 900 sequences per sample for bacteria and 950 for fungi. Rarefaction plots can be found in the Supplemental Material (Supplementary Figures S4–S7). Rarefication depths were chosen to ensure at least three replicates remained for each treatment. QIIME 2 artifacts were exported to R (version 3.6.3) (R Core Team, 2018) using the “qiime2R” package (Bisanz, 2018) to conduct statistical analysis using the “phyloseq” (McMurdie and Holmes, 2013) and “vegan” packages (Oksanen et al., 2019). Rarefied ASV tables and rooted trees were used for the community assembly analysis.

### Statistical Analysis

All statistical analyses were conducted in R (version 3.6.3) (R Core Team, 2018). One-way analysis of variance (ANOVA) was used to assess differences in soil abiotic parameters by depth. Assumptions of normality and homogeneity were assessed using the Shapiro–Wilk and Levene tests, respectively. Two-way ANOVA was used to assess differences in soil respiration rates by incubation temperature and depth. C:N ratios and respiration rates were log transformed for statistical analysis to improve assumptions of normality. Multiple comparisons were conducted using the Tukey’s HSD test to find which means were significantly different from one another.

Multivariate statistical analysis of community data was conducted using the ‘vegan’ package. Non-metric multidimensional scaling (NMDS) analysis using Bray–Curtis dissimilarity measure was used to evaluate differences in microbial community composition with depth and incubation temperature. Biplot vectors were added to the “initial” community NMDS plots using the “envfit()” function. Permutational multivariate analysis of variance (PerMANOVA) was conducted using the “adonis()” function to determine significant drivers of community composition for both “initial” and “post-thaw” communities. Depth and temperature were included as fixed factors in the PerMANOVA model and permutations were constrained by the permafrost core the samples originated from to account for random factors. PerMANOVA analysis is sensitive to significant differences in dispersion across groups (Anderson, 2006). Differences between group dispersion was assessed using the “betadisper()” function to calculate the average distance of samples to the group spatial median in multivariate space. Significance was assessed using a permutation test with the “permutest()” function conducting 999 permutations.

### Phylogenetic Signal and Null Modeling

The relative contribution of deterministic and stochastic assembly processes was determined for bacterial communities using phylogenetic turnover between samples and null models (Stegen et al., 2012, 2013). In order to infer ecological processes from phylogenetic information, there must be phylogenetic signal (Losos, 2008). Phylogenetic signal occurs when ecological similarity between species is related to their phylogenetic similarity. To test this assumption, phylogenetic signal was evaluated for GWC, pH, percent nitrogen, percent carbon, and the C:N ratio using the between-ASV difference in environmental optima and between-ASV phylogenetic distance. The environmental optima for each ASV was determined by calculating the abundance weighted mean for each environmental parameter tested (Stegen et al., 2012) using the “analogue” package (Simpson and Oksanen, 2020). This approximates the niche value of each abiotic variable for each ASV. A matrix of the between-ASV environmental optima differences was calculated using Manhattan distances for each abiotic variable. In addition to evaluating each abiotic variable individually, we calculated the combined ASV environmental optima using all of the abiotic variables measured. In brief, a matrix of the between-ASV combined environmental optima differences was calculated using the Euclidean distance measure of log normalized optima of all abiotic variables. The between-ASV phylogenetic distances were calculated using the “adephylo” package (Jombart and Dray, 2008). A mantel correlogram was generated using the “vegan” package by comparing each matrix of between-ASV environmental optima differences and the second matrix of between-ASV phylogenetic distances to evaluate the phylogenetic signal. Pearson’s correlation coefficients were calculated for fifty phylogenetic distance classes. The Mantel test statistic was determined using 999 permutations with progressive Holm-Bonferroni correction for multiple testing. Significant positive correlations indicate ecological similarity among ASVs is higher than expected by chance within the distance class (Borcard and Legendre, 2012). Alternatively, significant negative correlations indicate ASVs are more ecologically dissimilar than expected by chance (Borcard and Legendre, 2012).

Determination of assembly processes was only conducted for bacterial communities. The ITS region is appropriate for investigations of fungal community composition, but since it is not phylogenetically conserved it is inappropriate for phylogenetic modeling. Using the framework developed by Stegen et al. (2012, 2013), phylogenetic turnover between communities was quantified as the β-mean-nearest taxon distance (βMNTD) using the “picante” package (Kembel et al., 2010). This quantifies the mean phylogenetic distance between each member of a community and its closest relative in a second community. Null modeling of each community was then performed to create a distribution (*n* = 999) of βMNTD values representing a stochastic assembled community. For each iteration of the model, the tips of phylogeny were randomized and the βMNTD was recalculated. Deviations of the observed βMNTD from the null distribution were quantified in units of standard deviation of the null to generate the β-nearest taxon index (βNTI). Sample pairwise comparisons resulting in βNTI < −2 or βNTI > 2 indicates phylogenetic turnover is less than or greater than expected by chance, respectively, suggesting niche-based processes. Pairwise comparisons resulting in −2 > βNTI > 2 indicate stochastic processes structure turnover between the two communities. A modified Raup–Crick metric calculated using the Bray–Curtis dissimilarity measure (RC_bray_) was used to further differentiate the stochastic processes structuring the community (Chase et al., 2011). Table 1 summarizes the βNTI and RC_bray_ output values and how assembly processes were defined. The relative contribution of assembly processes was calculated by taking the fraction of pairwise comparisons demonstrating a given process and dividing by the total pairwise comparisons.

**TABLE 1:** Assembly processes and respective model conditions referenced from (Stegen et al., 2013).

Deterministic processes		Stochastic processes		
Homogeneous selection	Heterogeneous selection	Homogenizing dispersal	Dispersal limitation and drift	Drift alone
Environment constrains community composition through selection	Divergent environmental conditions result in each community having ecologically distinct members	High dispersal rates outweigh selective pressures	Movement of individuals is restricted	Population sizes fluctuate due to chance events
βNTI < −2	βNTI > 2	−2 < βNTI < 2		
–	–	RC_bray_ < −0.95	RC_bray_ > 0.95	−0.95 < RC_bray_ < 0.95

## Results

### Community Assembly Patterns Along Depth Profile

#### Soil Properties

**FIGURE 2:**
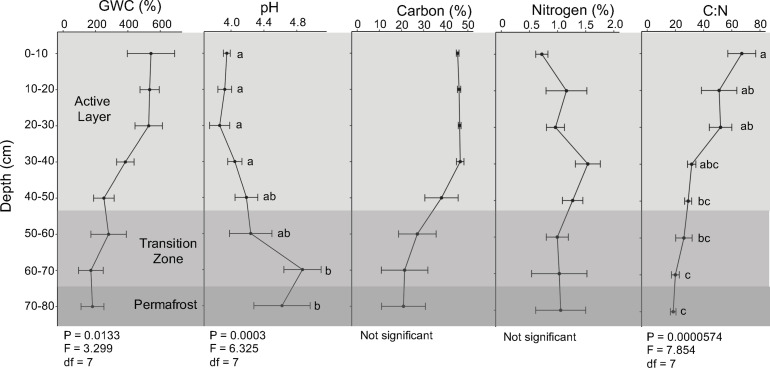
Abiotic parameters. Gravimetric water content (GWC), pH, total combustible carbon and nitrogen, and soil carbon to nitrogen ratio (C:N) of field soils along the depth profile. Points represent mean values, and error bars indicate ± standard error of the mean (*n* = 4). Means with the same letter are not significantly different within each abiotic parameter as determined by Tukey’s test (α = 0.05). C:N ratios were log transformed for statistical analysis.

Soil abiotic properties were determined at each depth. Average gravimetric water content exceeded 100% at all depths and was highest in the upper soil layers and decreased along the soil depth profile (Figure 2; ANOVA; *F* = 3.299, *P* = 0.0133). Soil pH was acidic (pH < 7) at all depths, with the upper soil layers being most acidic with an average pH of 3.9 (Figure 2; ANOVA; *F* = 6.325, *P* = 0.0003). Total combustible carbon was approximately 46% in the first 40 cm and decreased across 40 cm to 80 cm. Total combustible nitrogen fluctuated throughout the depth profile, ranging from 0.26% to 2.47%. The soil C:N ratio was approximately four times higher in the upper active layer compared to the permafrost and consistently decreased with depth (Figure 2; ANOVA; *F* = 7.854, *P* = 0.0000574).

#### Phylogenetic Signal

**FIGURE 3:**
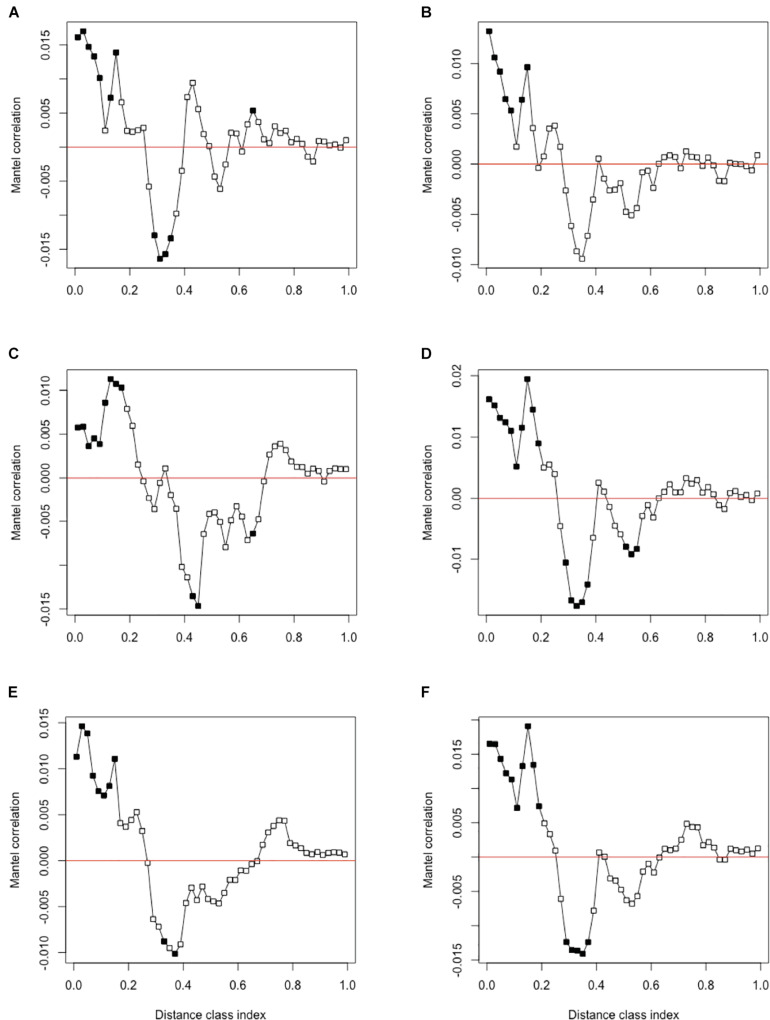
Mantel correlogram using Pearson’s correlation coefficient of between-ASV environmental optima and between-ASV phylogenetic distance for **(A)** pH, **(B)** % GWC, **(C)** % nitrogen, **(D)** % carbon, **(E)** C:N ratio, and **(F)** combined abiotic variables. Significant correlations (*P* < 0.05, solid circles) indicate phylogenetic signal in ASV ecological niche for the associated distance class. Significant positive correlations indicate that ecological similarity among ASVs is higher than expected by chance within the distance class. Alternatively, significant negative correlations indicate that ASVs are more ecologically dissimilar than expected by chance (Borcard and Legendre, 2012).

Phylogenetic signal was evaluated using Mantel correlograms comparing between-ASV environmental optima and between-ASV phylogenetic distances. All Mantel correlograms showed significant positive correlations across short phylogenetic distance (Figure 3). This relationship is consistent with other studies and indicates closely related species are more ecologically similar (Stegen et al., 2013; Wang et al., 2013; Dini-Andreote et al., 2015). Phylogenetic signal over short distances supports the use of βMNTD in determining the degree of ecological similarity since it calculates evolutionary distance between closely related species (Stegen et al., 2012; Wang et al., 2013). Significant negative correlations over intermediate phylogenetic distance classes were also observed. This indicates that ASVs at intermediate distances were more ecologically dissimilar than expected by chance. This further supports that using βMNTD is a robust method to infer ecological assembly processes since closely related taxa are most ecologically similar according to the Mantel correlogram results.

#### Microbial Community Dynamics Along the Depth Profile

**FIGURE 4:**
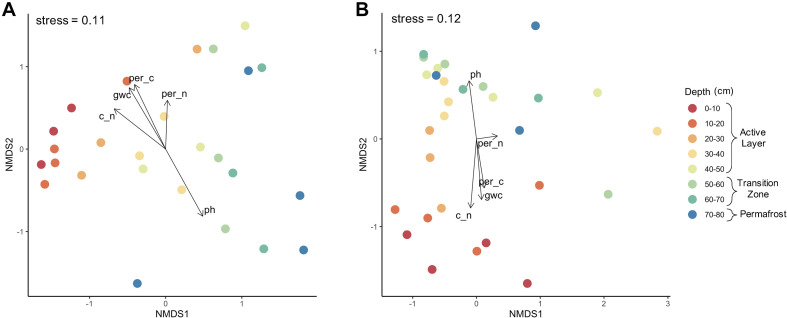
Microbial community composition by depth. Non-metric multidimensional scaling analysis using Bray–Curtis dissimilarity of **(A)** bacterial and **(B)** fungal communities along depth profile. Depth was a significant driver of community composition for both bacteria (PerMANOVA; *F* = 1.698, *R*^2^ = 0.3977, *P* = 0.0001) and fungi (PerMANOVA; *F* = 1.307, *R*^2^ = 0.3034, *P* = 0.0091).

Microbial community composition shifted with depth as indicated by NMDS analysis of bacterial and fungal communities using Bray-Curtis dissimilarity measures (Figure 4). Bacterial community composition clustered by depth for samples spanning the upper active layer: 0–10, 10–20, and 20–30 cm (Figure 4A). Fungal community composition also shifted with depth, however more clustering was observed within the deeper soils (60–70 cm and 70–80 cm) than was for bacterial communities. Differences in dispersion across depths were non-significant for both bacteria and fungi (Supplementary Table S1). For both bacteria and fungi, the core that the samples originated from was a significant driver of community composition (PerMANOVA; bacteria: *F* = 2.003, *R*^2^ = 0.2145, *P* = 0.003; fungi: *F* = 2.342, *R*^2^ = 0.2201, *P* = 0.0006) and was used to constrain permutations when investigating significant differences in community composition by depth. Depth was a significant driver of community composition for both bacteria (PerMANOVA; *F* = 1.698, *R*^2^ = 0.3977, *P* = 0.0001) and fungi (PerMANOVA; *F* = 1.307, *R*^2^ = 0.3034, *P* = 0.0091). Environmental vectors were fitted onto the NMDS ordinations and significance of the fitted vectors was assessed using permutation tests of the environmental variables. The five environmental variables measured significantly correlated with bacterial and fungal community compositions, with the exception of percent nitrogen for fungal communities (*P* > 0.05). Soil pH correlated strongly with NMDS axes, reflecting that pH was a strong driver of the observed differences in community composition (*P* < 0.003). Likewise, gravimetric water content, percent carbon, and the C:N ratio were also significant abiotic drivers (*P* < 0.01).

**FIGURE 5:**
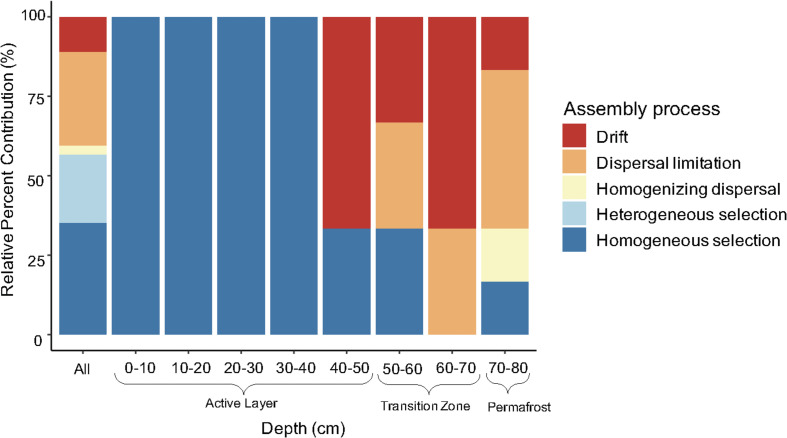
Relative contribution of assembly process structuring bacterial communities along the depth profile. Assembly processes were determined across all pairwise comparisons (“All”; *n* = 325) and within-group pairwise comparisons (*n* = 3 to 6) calculated from three to four biological replicates. Specifically, the 10–20 cm and 70–80 cm depth comparisons had *n* = 6 and the rest were *n* = 3.

Ecological processes structuring bacterial communities were determined using a phylogenetic null modeling approach. Assembly processes structuring bacterial communities changed along the depth profile (Figure 5). When looking at all possible pairwise comparisons in the dataset, results indicate that approximately 56% of the assembly process were deterministic with homogeneous selection being the most dominant (Figure 5). Heterogeneous selection was also observed, likely due to varying environmental conditions between samples in the pairwise comparisons. Homogeneous selection indicates constant selection pressures (e.g., environmental conditions) resulting in low turnover between communities while heterogeneous selection suggests there are differences between selection pressures resulting in a large amount of turnover between communities (Stegen et al., 2015). We determined the assembly processes governing community composition at each depth by including only the within depth pairwise comparisons. Bacterial communities in the upper to mid active layers (0–10 cm to 30–40 cm) were structured completely by homogenous selection (Figure 5). There was a shift in assembly processes between the 30–40 cm and 40–50 cm depths where an increase in stochastic processes was observed, specifically drift. The 60–70 cm depth was completely dominated by the stochastic processes drift and dispersal limitation. This depth represents the most recently thawed soils which transitioned from permafrost to active layer within the last fourteen years (Figure 1). Permafrost (70–80 cm) bacterial communities were also structured mostly by stochastic processes, particularly dispersal limitation which represented 50% of the assembly processes (Figure 5). Drift, homogenizing dispersal, and homogeneous selection were also important processes structuring permafrost bacterial communities.

### Community Assembly Patterns After Laboratory Thaw

#### Effect of Soil Depth and Incubation Temperature on Microbial Respiration

**FIGURE 6:**
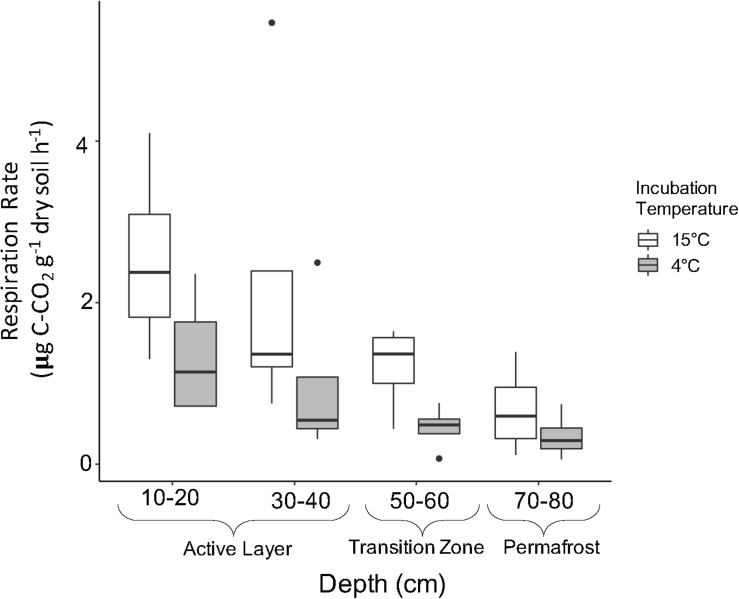
Soil respiration rates by depth of soils incubated at 15°C (white) and 4°C (gray). Respiration rates were determined using stabilized rates from day 20 to 193 of incubation. Boxplots show median value as a solid line and upper and lower quartiles at the top and bottom of the boxes, respectively. Whiskers and points indicate the extent of the data. Respiration rates were log transformed for statistical analysis. Two-way ANOVA indicated significant effects of temperature (*F* = 7.683, *P* = 0.01060) and depth (*F* = 5.134, *P* = 0.00695) on respiration, but no combined effect.

Microbial respiration was monitored throughout the incubation to assess general microbial activity and release of CO_2_ with increased temperature. Respiration rates significantly decreased with depth (Figure 6; Two-way ANOVA; *F* = 5.134, *P* = 0.00695) where the 10–20 cm rate was four times greater than the 70–80 cm rate at both temperatures. Average respiration rates were significantly higher in soils incubated at 15°C compared to 4°C (Figure 6; Two-way ANOVA; *F* = 7.683, *P* = 0.01060). In general, average respiration was two to three times higher at 15°C compared to 4°C.

**FIGURE 7:**
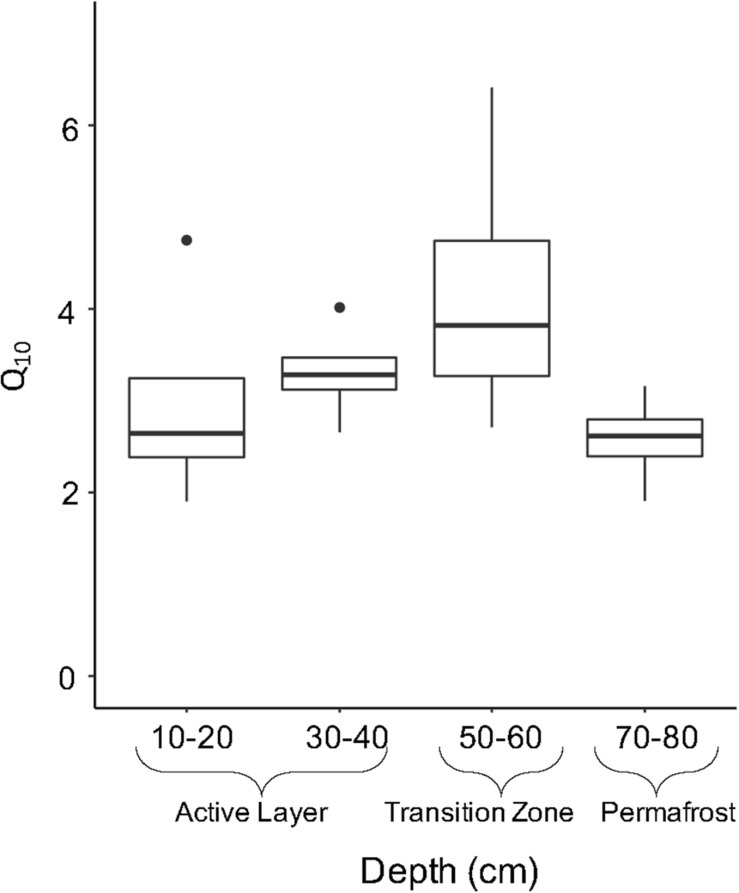
Temperature sensitivity (*Q*_10_) by soil depth. *Q*_10_ is an indicator of the temperature sensitivity of respiration and were calculated from CO_2_ respiration rates at 15°C and 4°C. Boxplots show median value as a solid line and upper and lower quartiles at the top and bottom of the boxes, respectively. Whiskers and points indicate the extent of the data.

Temperature sensitivity (*Q*_10_) of microbial respiration in soil samples was calculated to determine the factor by which heterotrophic respiration increases when temperature is increased by 10°C for these samples. No significant difference in *Q*_10_ values were observed between soil layers. The upper active layer (10–20 cm) and permafrost soils (70–80 cm) showed similar average Q_10_ values of 2.88 ± 0.59 and 2.49 ± 0.25, respectively (Figure 7). The transition zone soils showed the highest temperature sensitivity of all depths with an average of 4.05 ± 0.77 (Figure 7).

#### Microbial Community Dynamics With Incubation Conditions

**FIGURE 8:**
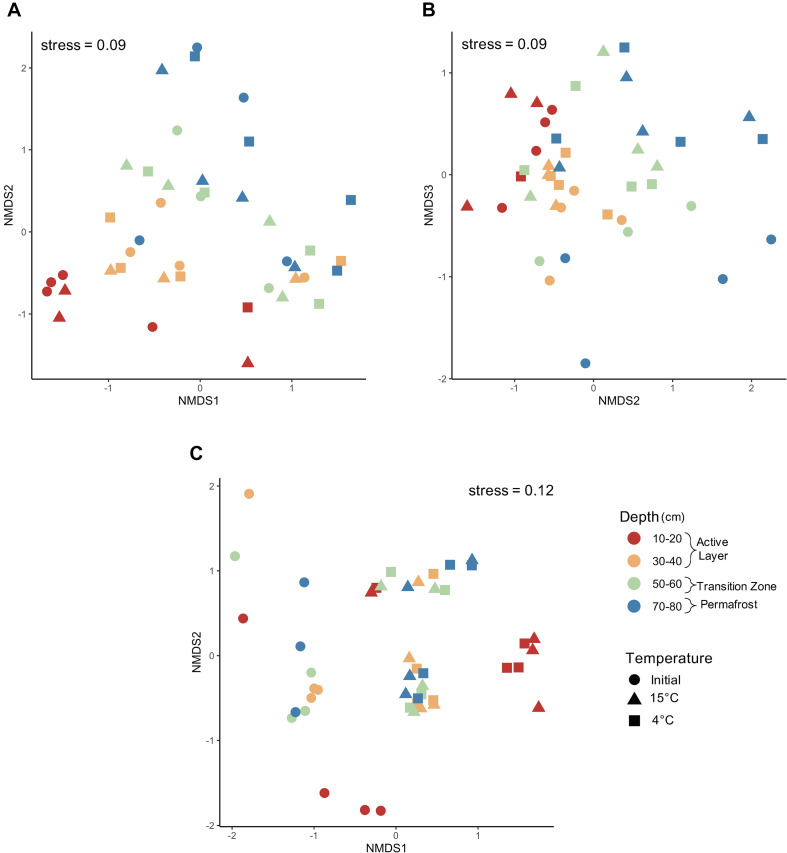
Microbial community composition pre- and post-incubation. Non-metric multidimensional scaling analysis using Bray–Curtis dissimilarity community composition of **(A)** bacteria axes 1–2, **(B)** bacteria axes 2–3, and **(C)** fungi axes 1–2 before and after incubation at 4°C and 15°C. Bacterial communities were significantly structured by both depth and temperature (PerMANOVA; depth: *F* = 2.864, *R*^2^ = 0.1876, *P* = 0.0001; temperature: *F* = 1.422, *R*^2^ = 0.0621, *P* = 0.0027). Additionally, fungal communities were significantly structured by both depth and temperature (PerMANOVA; depth: *F* = 2.075, *R*^2^ = 0.1155, *P* = 0.0001; temperature: *F* = 3.489, *R*^2^ = 0.1332, *P* = 0.0001).

Changes to the microbial community with thaw were assessed by comparing pre- and post-incubation communities at 4°C and 15°C. The laboratory incubation allowed us to examine the direct effect of temperature change on microbial communities found in each of the soil depth layers. Non-metric multidimensional scaling (NMDS) analysis using Bray-Curtis dissimilarity measures was used to determine if increased temperature resulted in changes to microbial community composition along the depth profile (Figure 8). Differences in group dispersion were non-significant for depth and temperature (Supplementary Table S1). For both bacteria and fungi, core was a significant driver of community composition (Supplementary Figures S2, S3; PerMANOVA; bacteria: *F* = 2.842, *R*^2^ = 0.1833, *P* = 0.0001; fungi: *F* = 2.414, *R*^2^ = 0.1440, *P* = 0.0001) and was used to constrain permutations when investigating community composition by depth and temperature. A three-dimensional NMDS solution was found (stress value < 0.2) for bacterial community structure and indicated strong clustering by depth with the deeper soils being less clustered compared to the active layer samples (Figures 8A,B). When looking at NMDS axes 2 and 3, the 50–60 cm and 70–80 cm depths grouped by pre- and post-incubation (Figure 8B). Both depth and temperature were significant drivers of bacterial community composition (PerMANOVA; depth: *F* = 2.864, *R*^2^ = 0.1876, *P* = 0.0001; temperature: *F* = 1.422, *R*^2^ = 0.0621, *P* = 0.0027); however, depth seemed to explain more variation in community composition compared to temperature. Depth and temperature were also significant drivers of fungal community composition (PerMANOVA; depth: *F* = 2.075, *R*^2^ = 0.1155, *P* = 0.0001; temperature: *F* = 3.489, *R*^2^ = 0.1332, *P* = 0.0001) with strong clustering observed by temperature (Figure 8C). In general, the pre- and post-incubation 10-20 cm samples clustered separately from the other three depths after incubation. The three samples that did not follow this pattern were all from the same core which clustered somewhat on its own (Supplementary Figure S3).

**FIGURE 9:**
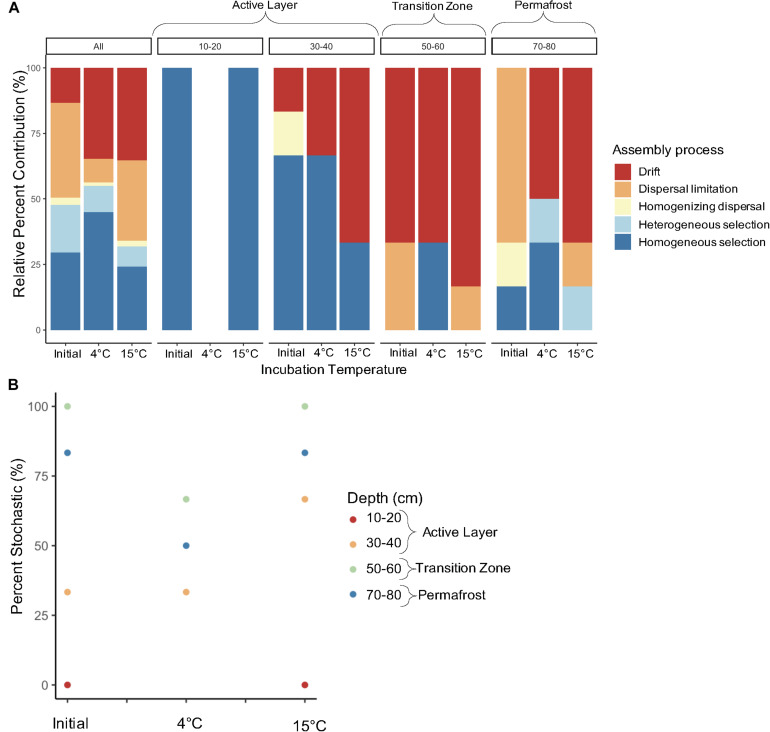
Relative contribution of assembly process structuring bacterial communities pre- and post-incubation at 4°C and 15°C. The **(A)** relative percent contribution of each assembly process and **(B)** percent stochastic assembly are shown by depth. Assembly processes were determined across all pairwise comparisons (“All”, *n* = 861) and within-group pairwise comparisons (*n* = 3 to 6) calculated from three to four biological replicates. The 10–20–4°C treatment only had one replicate and therefore pairwise comparisons for that treatment could not be made. Note that the 10–20–4°C data point on **(B)** is missing.

We investigated the direct effect of increased temperature on assembly processes structuring microbial communities in along the depth profile. Dominant assembly processes were assessed to determine how assembly may differ based on near-term versus long-term thaw conditions. When looking at the effect of temperature on assembly of all depths combined, there was a slight increase in deterministic assembly between the initial and 4°C samples, but incubation at 15°C led to a 16% increase in stochastic assembly (Figure 9A). Laboratory incubation resulted in an increase in drift at both temperatures when all comparisons were considered. Varying shifts in assembly processes with incubation temperature were observed within each depth (Figure 9A). Processes structuring bacterial communities in the 10-20 cm layer remained deterministic after incubation (Figure 9). Only one of the 4°C samples for the 10–20 cm layer made it through quality control of the sequencing data and therefore pairwise comparisons could not be made for this treatment. An increase in stochastic processes was observed with warmer incubation temperature in the 30–40 cm depth. Within this depth, incubation at 15°C resulted in an increase from 33% to 66% stochastic assembly with drift being the dominant process (Figure 9). Both the 50–60 cm and 70–80 cm depths showed an increase in deterministic assembly from initial to 4°C samples and then a shift back to mostly stochastic assembly at 15°C (Figure 9B). The 50–60 cm depth was dominated by drift both pre- and post-incubation (Figure 9A). Permafrost (70–80 cm) bacterial communities were structured mainly by dispersal limitation in the frozen state, but upon thaw at 4°C and 15°C there was a shift to drift (Figure 9A). Heterogenous selection also emerged as an important deterministic process post-thaw for permafrost samples. Dispersal limitation and homogenizing dispersal were shown to be important processes structuring bacterial communities in the 50–60 cm and 70–80 cm samples after incubation in isolated microcosms (Figure 9A). However, the relative percent contribution of these two processes was less than that shown for the initial samples in both cases. The presence of these dispersal-based processes after incubation in isolated containers reflects their initial importance in structuring the communities in the field.

## Discussion

Microbial communities are structured by a combination of deterministic and stochastic processes, the outcome of which may have implications for how the community processes organic matter (Delgado-Baquerizo et al., 2016; Trivedi et al., 2016). Functional, taxonomic, and phylogenetic beta diversity are closely correlated in soil microorganisms (Fierer et al., 2012). Furthermore, Martiny et al. (2013) found that 93% of functional traits they investigated were non-randomly distributed across phylogeny with most traits being shared only among phylogenetically similar taxa. Given this relationship between phylogeny and function, the use of phylogenetic approaches to investigate drivers of community structure could be used to predict how function might change due to disturbance. Our findings suggest there is a shift toward stochastic assembly immediately after thaw, particularly the influence of drift, except in upper active layer soils. The active layer soils that have been seasonally thawed for many years exhibited mainly deterministic assembly indicating the importance of selection under long-term thaw. Understanding the processes controlling community reorganization after permafrost thaw will improve our ability to develop a robust framework to predict the ecological impact of permafrost thaw.

### Permafrost Thaw Resulted in Increased Stochastic Assembly

Assembly processes structuring bacterial communities shifted along the depth profile. Transition zone soil at the active layer-permafrost interface represents the most recently thawed soils, and the active layer soils have been seasonally thawed for a longer period of time. Permafrost and transition zone soils were dominated by stochastic community assembly, but deterministic processes also played a role in permafrost soils which is unsurprising given that stochastic and deterministic processes work in combination to influence community composition (Vellend, 2010). In intact permafrost, bacterial communities were structured mainly by dispersal limitation, but homogenizing dispersal, drift, and homogenizing selection also played a role in structuring permafrost communities. Bottos et al. (2018) also found dispersal limitation as the dominant process structuring permafrost bacterial communities across a permafrost transect in Central Alaska and found contributions of homogenizing dispersal and homogenous selection. Homogenizing dispersal describes high dispersal rates that outweigh selection pressures and is often seen in combination with dispersal limitation, but generally one is much more dominant than the other (Bottos et al., 2018; Tripathi et al., 2019). Homogenous selection occurs when a homogenous environment selects for ecologically similar taxa, which demonstrates that the permafrost environment is spatially homogenous and imposes limitations on the phylogenetic and functional diversity of the system. Our hypothesis that permafrost soils would be dominated by selection due to necessary survival strategies to tolerate adverse conditions was not supported. Instead, the physical constraints on dispersal were the dominant ecological process governing permafrost bacterial community structure. Although dispersal is often described as a stochastic process, some argue dispersal can be influenced by both stochastic and deterministic factors (reviewed in Nemergut et al., 2013; Zhou and Ning, 2017). In the case of permafrost, dispersal rates are partly dependent on the frozen environmental conditions and metabolic state of community members suggesting dispersal limitation may be partly deterministic. Frozen soil restricts the passive dispersal of microbes and cold temperature promotes a high degree of dormancy in the community therefore limiting their active dispersal abilities.

The transition zone soils, which most recently transitioned from permafrost to seasonally thawed active layer soils, were completely dominated by stochastic assembly processes which supported our hypothesis. Drift (random fluctuations in population sizes due to chance events) was the dominant process in the transition zone soils which indicates there may have been a great amount of turnover and extinction after thaw. Drift is an important process when local community size is small, selection is weak, and populations are present in low-abundances which could lead to local extinction (Chase and Myers, 2011; Nemergut et al., 2013). We did not measure population size in our study; however, in the case of permafrost thaw, it likely decreases due to drastic changes in the local environment immediately after thaw resulting in the closing of permafrost-specific niches, affecting individuals’ ability to survive in the new environment. Future work should measure population size when assessing the contribution of assembly processes, particularly in the case of drift. Dispersal limitation also contributed to community assembly within transition zone soils suggesting dispersal between soil layers is still somewhat restricted immediately after thaw. The large influence of stochastic processes likely contributed to the observed variation of community composition in the permafrost and transition zone because the randomness of these processes allows for different composition trajectories (Fukami, 2015). These results are consistent with findings from Monteux et al. (2018) where the authors also observed more variation in community composition of the deeper soils at the same field site. Increases in stochastic assembly have been observed with increased depth in permafrost systems, particularly the influence of drift (Tripathi et al., 2018, 2019). Tripathi et al. (2019) also found dispersal limitation was highest in the deepest soil horizon in their study.

Active layer soils were dominated by deterministic assembly processes, suggesting that as time since thaw increases, there is a shift from stochastic to deterministic assembly. Upper active layer bacterial communities were completely dominated by homogenous selection indicating that there was less turnover between active layer communities than expected by chance. Tripathi et al. (2019) also found homogeneous selection was the dominant process structuring upper active layer bacterial communities which they attributed to minimal variation in physiochemical properties within this layer. We also observed little variation in pH and % carbon. Feng et al. (2020) observed an increase in deterministic assembly in permafrost-affected surface soils under long term warming which is consistent with our findings that the longer soils were thawed, the less stochastic assembly was observed. When investigating bacterial community assembly along a natural thaw gradient, Mondav et al. (2017) found there was a slight increase in deterministic assembly moving from intact to degraded permafrost sites. This further supports the finding that soils under long-term thaw transition from stochastic to deterministic assembly. Our hypothesis that the upper active layer would be dominated by deterministic assembly was supported. We observed assembly processes in the transition zone soils were largely stochastic and as time since thaw increased up the depth profile, the contribution of deterministic processes also increased. Our results support the framework developed by Ferrenberg et al. (2013) that community assembly immediately after a disturbance is likely to be stochastic and as succession progresses deterministic processes begin structuring communities. However, one drawback of using depth as a proxy of “time since thaw” is that it is difficult to tease apart the influence of soil physiochemical properties and the time, but these factors are intimately linked with permafrost thaw making this approach a reasonable approximation of *in situ* processes.

### Laboratory Induced Thaw Increased Relative Contribution of Drift

Our hypothesis that assembly would be more stochastic at all depths after lab-induced thaw compared to *in situ* assembly, due to the short successional time frame after the disturbance, was not supported. Instead, we observed similar percent stochasticity at pre- and post-incubation at 15°C. The permafrost and transition zone soils showed a slight decrease in stochastic assembly from pre- to post-incubation at 4°C. However, the importance of drift in structuring communities after thaw—a key finding from the field study—was confirmed in the laboratory incubations. The βNTI values from pairwise comparisons of all samples showed an increase in drift of 13% to 35% after incubation. Even larger increases in the relative contribution of drift were observed for the permafrost (70–80 cm) and lower active layer (30–40 cm) samples with the 15°C incubation temperature yielding the highest percent drift. The transition zone (50–60 cm) was initially dominated by drift, which only increased with incubation at 15°C. The observation that there is a large increase in drift upon thaw is consistent with the finding from the field study where a large increase in drift was seen in the transition zone. This suggests that laboratory incubation studies simulating permafrost thaw are good at showing the immediate effects of thaw, but that to understand the long-term effects longer incubations may be necessary. Given that the samples used in the incubation study thawed in transit, community trajectories might have been influenced by populations that emerged at room temperature. However, the large amount of turnover (i.e., drift) observed even after 193 days of incubation suggests these community members may not have had a greater fitness advantage after thaw.

When drift dominates, it may be difficult to predict the resulting community structure and function due to the random fluctuations in community member abundance. This makes inference of long-term effects of community change with thaw difficult using short-term lab studies (Elberling et al., 2013; Knoblauch et al., 2013). Lab incubations, at least those done in the short term, may not completely capture shifts in assembly at mid to late succession. Furthermore, laboratory studies usually do not incorporate the interactions between active layer and permafrost microbes, and since dispersal limitation is an important process structuring transition zone and permafrost communities, this should be considered. Experiments are needed to investigate permafrost thaw under realistic conditions where natural dispersal and seasonal changes can occur.

### The Influence of Assembly Dynamics on Community Function

Disturbance events may alter ecosystem functioning when the disturbance results in a change in community composition—a common occurrence in microbial systems (Allison and Martiny, 2008). Different assembly processes can result in communities with varying compositions that harbor a distinct suite of functional traits and ultimately influence ecosystem functions (Knelman and Nemergut, 2014). Furthermore, assembly processes can influence community function due to fitness of individuals and their ability to contribute toward biogeochemical functions versus spending energy on survival and maintenance (Graham and Stegen, 2017). Some argue that information on microbial community composition is not necessary to predict how they will function (Schimel, 2001), however this is based on the assumptions that community assembly is controlled by selection (Nemergut et al., 2013) and that traits are always selected by the environment. Microbial communities structured by stochastic processes likely lack a direct link to environmental parameters and predicting functional outcomes based on environmental conditions alone may be difficult (Nemergut et al., 2013). While the synthesis by Allison and Martiny (2008) found that changes in community composition resulted in changes in function and the simulation modeling by Graham and Stegen (2017) show assembly processes can influence biogeochemical function, the assembly-function link proposed by Nemergut et al. (2013) is largely untested by experimental data.

Stochastic assembly in the months after permafrost thaw may result in decreased fitness of individuals leading to greater release of carbon from the soil. We speculate that permafrost thaw leads to a great amount of physiological stress for individuals in the community. This shift in environmental conditions may result in a stochastically assembled community dominated by drift and comprised of unfit taxa that invest most of their energy to survival and maintenance rather than biogeochemical processes (Schimel et al., 2007). Alternatively, increased CO_2_ emissions could result due to fast-growing taxa proliferating after thaw. In either instance, unfit and fast-growing taxa are thought to be less efficient than their counterparts (reviewed in Molenaar et al., 2009; Roller and Schmidt, 2015) resulting in more carbon released as CO_2_ in relation to carbon assimilated into biomass (Manzoni et al., 2012).

To test the link between assembly processes and carbon emission from thawing permafrost, we measured CO_2_ respiration and compared it with the dominant assembly processes at each soil layer. We observed significant differences in microbial respiration along the soil depth profile and with incubation temperature. The transition zone soil showed increased temperature sensitivity (Q_10_) indicating the respiration rate of these soils was most positively affected by an increase in temperature; however, this was not significantly higher than that of the other soil depths. Our results do not indicate there is a direct link between carbon emission from microbial respiration and assembly processes structuring communities. Respiration is conserved across many microbial taxa and shifts in assembly processes are less likely to affect broad functions that are redundant in a community. More specific traits, such as ability to use different carbon substrates, are less phylogenetically conserved and are only shared between closely related taxa (Martiny et al., 2013). In the case of these more specific functions, we would expect to see a relationship between assembly dynamics and functional outcomes. While our study suggests that differences in assembly processes do not have a direct link with CO_2_ emissions, future research should investigate the assembly-function link over a broad suite of functions to better understand the role of these recently disturbed communities in the permafrost-climate feedback.

## Conclusion

The objective of this study was to determine the ecological processes structuring microbial communities in permafrost and active layer soils. We found that depth was a strong driver of microbial community composition in both pre- and post-incubation soils. Permafrost and transition zone soils were dominated by stochastic processes with drift and dispersal limitation being the main processes controlling bacterial community structure. Deterministic processes, specifically homogeneous selection, were dominant in the active layer soils. Thawed conditions in both the field study and lab incubation increased the relative contribution of stochastic processes, particularly the importance of drift, in structuring communities. This was potentially the result of closing and opening of niches that lead to a large amount of turnover in community composition after thaw, particularly those with small population sizes. The laboratory incubation study reflected community assembly dynamics immediately after thaw, but precludes understanding the effect of assembly over long-term succession post-thaw. Our results suggest stochastic assembly immediately after thaw did not result in increased carbon emissions; however, future work should investigate the relationship between assembly dynamics and functional outcomes, particularly across the range of broad to specific functions.

## Data Availability Statement

The datasets presented in this study can be found in online repositories. The names of the repository/repositories and accession number(s) can be found below: https://www.ncbi.nlm.nih.gov/, PRJNA657840. Code used for modeling and statistical analyses is available at GitHub (https://github.com/sljarvis2).

## Author Contributions

SD, JE, RB, AG, and WT contributed to the experimental design. SM, ED, and MJ contributed to the field sampling design. MJ contributed to the active layer thickness data. SD and RB conducted the permafrost sampling and core processing. SD performed the experiments and analyzed the data. JE provided the experiment oversight and contributed to the data interpretation. SD wrote the first draft of the manuscript. All authors contributed to the manuscript revision, and read and approved the submitted version.

## Conflict of Interest

The authors declare that the research was conducted in the absence of any commercial or financial relationships that could be construed as a potential conflict of interest.
