# Supplementary material for: The Transition From Stochastic to Deterministic Bacterial Community Assembly During Permafrost Thaw Succession
Source: Front Microbiol. 2020 Nov 13;11:596589. doi: 10.3389/fmicb.2020.596589 (PMC7691490; doi:10.3389/fmicb.2020.596589)
Supplement: Supplementary file 1 [file Data_Sheet_1.docx]

# Supplemental Material

DNA Extraction Protocol Modifications

Modified protocol for Qiagen DNeasy DNA PowerSoil Kit to increase DNA yield.

1. Two blanks (tubes without soil) are included in each extraction.
2. Add 0.25 g of soil sample into the **PowerBead Tubes** provided.
3. Gently vortex **PowerBead Tubes** to mix.
4. Check **Solution C1**. If **Solution C1** has precipitated, heat solution to 60°C until dissolved before use. Add 60 μl of **Solution C1** to each **PowerBead Tube** and invert several times or vortex briefly.
5. Place tubes in the bead beater plate, and secure plate in the bead beater. Shake for 4 minutes.
6. Centrifuge **PowerBead Tubes** at 10,000 x g for 1 minute at room temperature.
7. Transfer all supernatant to a clean **2 ml Collection Tube** (provided). Expect ~ 750 μl of supernatant. Supernatant may still contain some soil particles.
8. Add 250 μl of **Solution C2** (stored in fridge) and vortex for 5 seconds. Incubate at 4°C for 5 minutes.
9. Centrifuge the tubes at 10,000 x g for 1 minute at room temperature.
10. Avoiding the pellet, transfer all (~1000 μl) supernatant into a clean **2 ml Collection Tube** (provided).
11. Add 330 μl of **Solution C3** (stored in fridge) to each tube and vortex briefly. Incubate at 4°C for 5 minutes
12. Centrifuge the tubes at 10,000 x g for 1 minute at room temperature.
13. Avoiding the pellet, transfer all (~1100 μl) of supernatant into a clean **2 ml Collection** **Tube** (provided). Split this volume between two tubes (~550 μl each). Use 200 μl tips.
14. Shake to mix **Solution C4** before use. Add 880 μl **Solution C4** into each tube of supernatant. Vortex for 5 seconds.
15. Load the **Spin Filter** onto the VacConnectors on the Qiagen manifold.
16. With the manifold vacuum off, load approximately 675 μl onto each **Spin Filter**. Turn on the manifold vacuum until the loaded supernatant has been pulled through each spin filter, then turn off the manifold vacuum (should be no more than 5 seconds). Repeat this step until all of the supernatant has been loaded and vacuumed through the spin filter. Leave the spin filters attached to the manifold. Note: A total of 4-5 loads for each sample processed are required.
17. Load 500 μl of **Solution C5** onto each **Spin Filter**. Turn on the manifold vacuum until the loaded solution has been pulled through each spin filter, then turn off the manifold vacuum.
18. Remove each **Spin Filter** from the manifold and place them back in their original collection tubes. Centrifuge the **Spin Filters** for a dry spin at room temperature for 1 minute at 10,000 x g.
19. Carefully place **Spin Filter** in a clean **2 ml Collection Tube** (provided). Avoid splashing any **Solution C5** onto the **Spin Filter**.
20. Add 100 μl of **Solution C6** to the center of the white filter membrane and let **Solution C6** sit on filter for 5 minutes at room temperature.
21. Centrifuge at room temperature for 1 minute at 10,000 x g.
22. Discard the **Spin Filter**. The DNA in the tube is now ready for any downstream application. Store DNA at -20°C.

Sequencing Primers

**Table S1.** Amplicon sequencing primers

| Target | Primer | Primer sequence | Reference |
| --- | --- | --- | --- |
| 16S rRNA | 515F-806R | Forward: GTGYCAGCMGCCGCGGTAA  Reverse: GGACTACNVGGGTWTCTAAT | (Apprill et al., 2015; Parada et al., 2016) |
| ITS | ITS1f-ITS2 | Forward: CTTGGTCATTTAGAGGAAGTAA  Reverse: GCTGCGTTCTTCATCGATGC | (White et al., 1990) |

Supplemental Figures

**
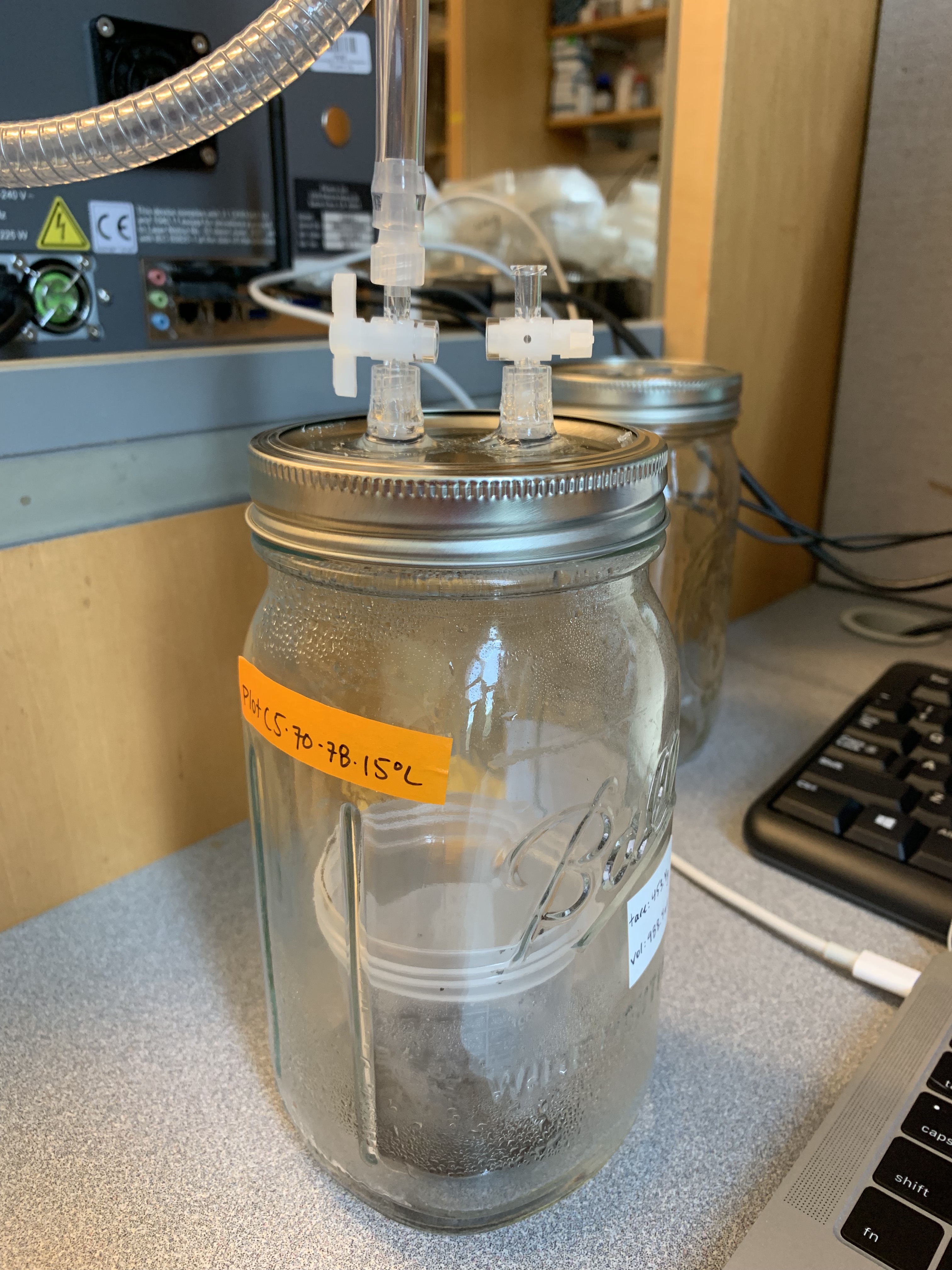
**

Figure S1. Photo of example incubation jar containing specimen cup with soil sample and valve lid for measurements.

*
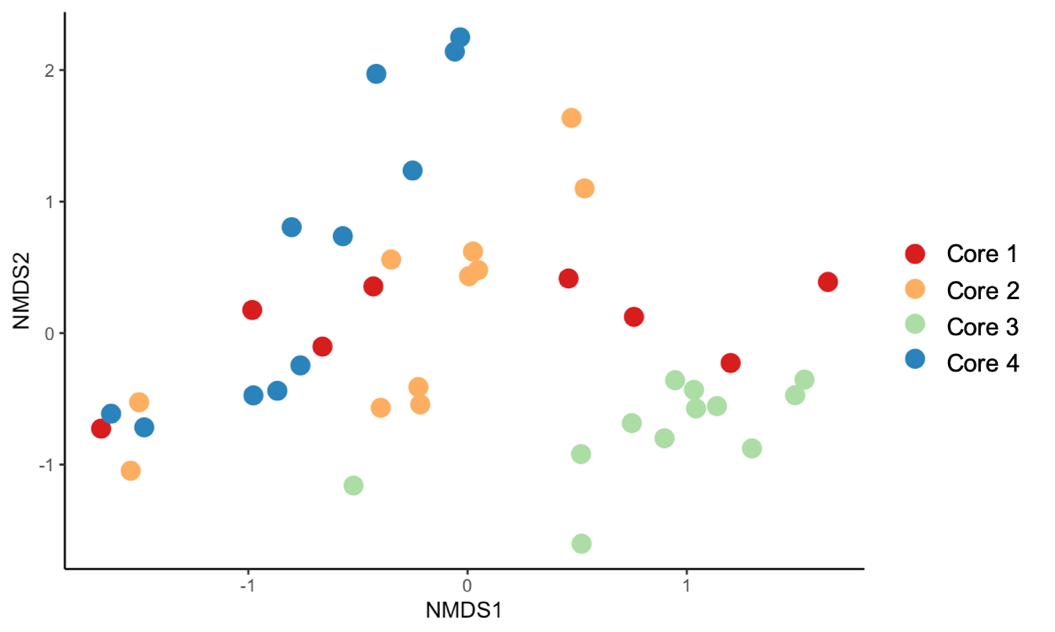
*

Figure S2. Bacterial community composition pre- and post-incubation. Non-metric multidimensional scaling analysis based on Bray-Curtis dissimilarity community composition of bacteria colored by core the samples originated from.

*
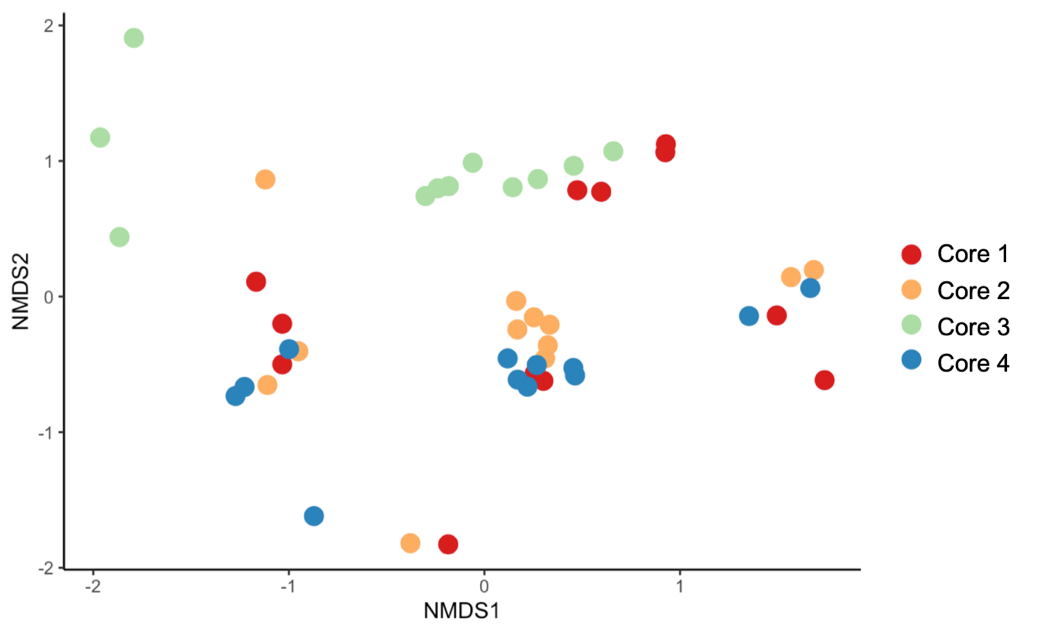
*

Figure S3. Fungal community composition pre- and post-incubation. Non-metric multidimensional scaling analysis based on Bray-Curtis dissimilarity community composition of fungi colored by core the samples originated from.


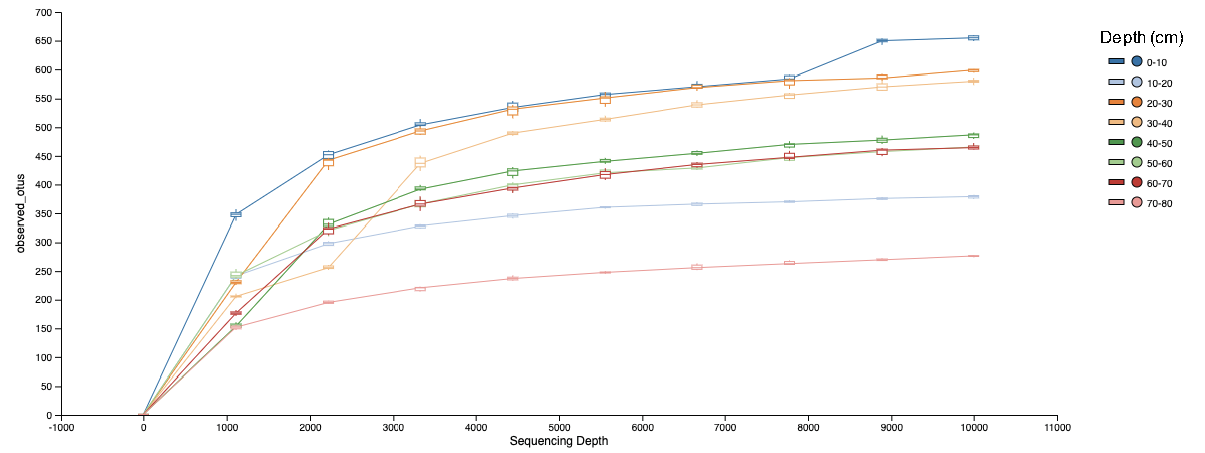


Observed ASVs

Figure S4. Rarefaction plot of bacterial sequencing data along the soil depth profile. Samples were rarefied to 2500 sequences per sample.


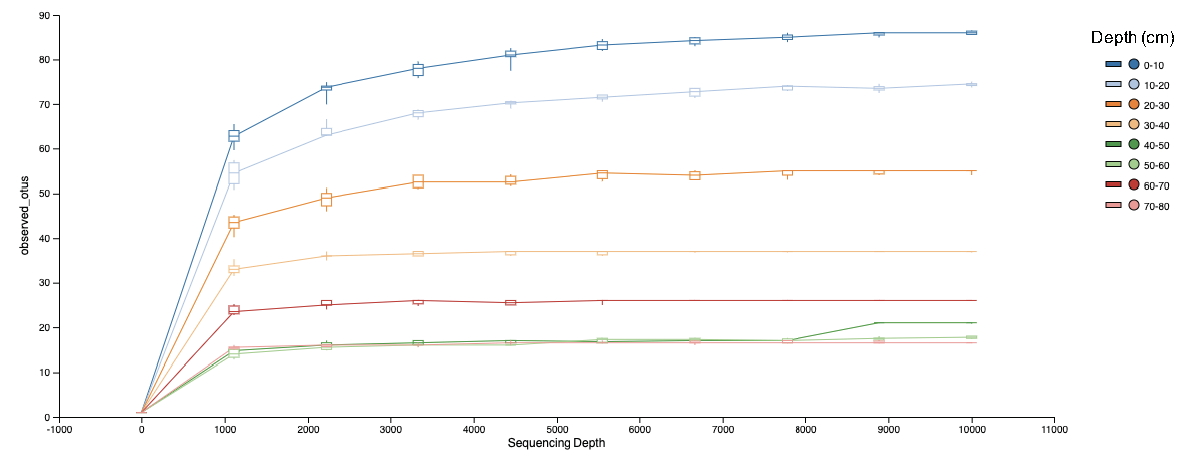


Observed ASVs

Figure S5. Rarefaction plot of fungal sequencing data along the soil depth profile. Samples were rarefied to 950 sequences per sample.


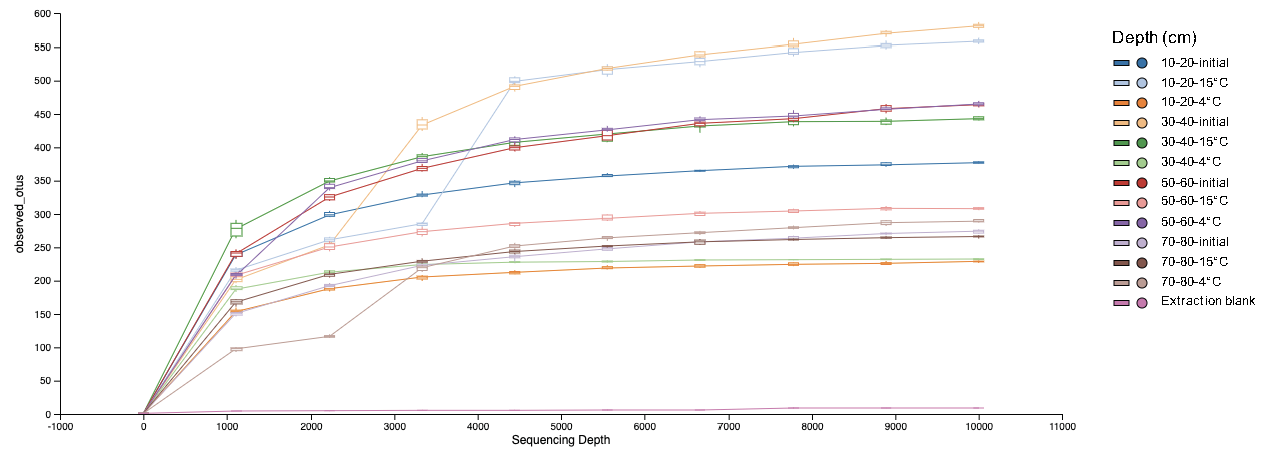


Observed ASVs

Figure S6. Rarefaction plot of bacterial sequencing data from soil incubation study. Samples were rarefied to 900 sequences per sample.


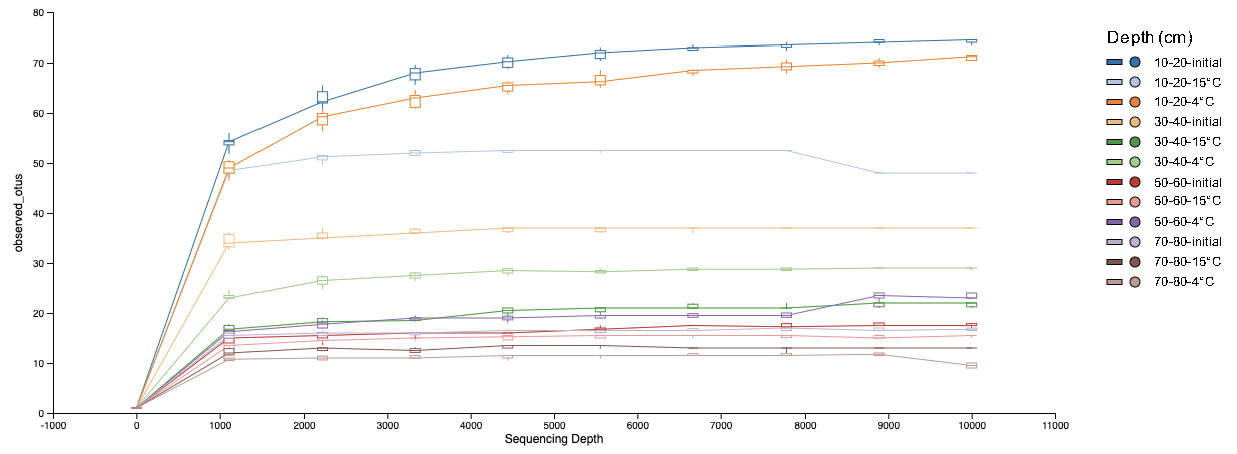


Observed ASVs

Figure S7. Rarefaction plot of fungal sequencing data from soil incubation study. Samples were rarefied to 950 sequences per sample.

Table S1. Differences between group dispersion for microbial communities along the depth profile and soil incubation.

| **Sample set** | **Df** | **F** | **P-value** |
| --- | --- | --- | --- |
| Depth profile - bacteria | 7 | 0.4919 | 0.851 |
| Depth profile - fungi | 7 | 0.2192 | 0.974 |
| Incubation - bacteria | 3 | 1.5402 | 0.224 |
| Incubation - fungi | 3 | 0.3037 | 0.814 |
